# A Comprehensive Cytogenetic Analysis of Several Members of the Family Columbidae (Aves, Columbiformes)

**DOI:** 10.3390/genes11060632

**Published:** 2020-06-08

**Authors:** Rafael Kretschmer, Ivanete de Oliveira Furo, Anderson José Baia Gomes, Lucas G. Kiazim, Ricardo José Gunski, Analía del Valle Garnero, Jorge C. Pereira, Malcolm A. Ferguson-Smith, Edivaldo Herculano Corrêa de Oliveira, Darren K. Griffin, Thales Renato Ochotorena de Freitas, Rebecca E. O’Connor

**Affiliations:** 1 School of Biosciences, University of Kent, Canterbury CT2 7NJ, UK; lgk3@kent.ac.uk (L.G.K.); d.k.griffin@kent.ac.uk (D.K.G.);; 2 Departamento de Genética, Universidade Federal do Rio Grande do Sul, Porto Alegre 91509-900, Brazil; thales.freitas@ufrgs.br; 3 Instituto de Ciências Biológicas, Universidade Federal do Pará, Belém 66075-110, Brazil; ivanetefuro100@gmail.com; 4 Laboratório de Cultura de Tecidos e Citogenética, SAMAM, Instituto Evandro Chagas, Ananindeua 67030-000, Brazil; ehco@ufpa.br; 5 Instituto Federal do Pará, Abaetetuba 68440-000, Brazil; anderson.gomes@ifpa.edu.br; 6 Laboratório de Diversidade Genética Animal, Universidade Federal do Pampa, São Gabriel 97300-162, Brazil; ricardogunski@unipampa.edu.br (R.J.G.); analiagarnero@unipampa.edu.br (A.d.V.G.); 7 Animal and Veterinary Research Centre (CECAV), University of Trás-os-Montes and Alto Douro (UTAD), 5000-801 Vila Real, Portugal; jorgecpereira599@gmail.com; 8 Cambridge Resource Centre for Comparative Genomics, University of Cambridge Department of Veterinary Medicine, Cambridge CB3 0ES, UK; maf12@cam.ac.uk; 9 Instituto de Ciências Exatas e Naturais, Universidade Federal do Pará, Belém 66075-110, Brazil

**Keywords:** birds, doves and pigeons, evolution, genome organization, macrochromosomes, microchromosomes

## Abstract

The Columbidae species (Aves, Columbiformes) show considerable variation in their diploid numbers (2n = 68–86), but there is limited understanding of the events that shaped the extant karyotypes. Hence, we performed whole chromosome painting (wcp) for paints GGA1-10 and bacterial artificial chromosome (BAC) probes for chromosomes GGA11-28 for *Columbina passerina*, *Columbina talpacoti*, *Patagioenas cayennensis*, *Geotrygon violacea* and *Geotrygon montana*. *Streptopelia decaocto* was only investigated with paints because BACs for GGA10-28 had been previously analyzed. We also performed phylogenetic analyses in order to trace the evolutionary history of this family in light of chromosomal changes using our wcp data with chicken probes and from *Zenaida auriculata*, *Columbina picui*, *Columba livia* and *Leptotila verreauxi*, previously published. G-banding was performed on all these species. Comparative chromosome paint and G-banding results suggested that at least one interchromosomal and many intrachromosomal rearrangements had occurred in the diversification of Columbidae species. On the other hand, a high degree of conservation of microchromosome organization was observed in these species. Our cladistic analysis, considering all the chromosome rearrangements detected, provided strong support for *L*. *verreauxi* and *P*. *cayennensis*, *G*. *montana* and *G*. *violacea*, *C*. *passerina* and *C*. *talpacoti* having sister taxa relationships, as well as for all Columbidae species analyzed herein. Additionally, the chromosome characters were mapped in a consensus phylogenetic topology previously proposed, revealing a pericentric inversion in the chromosome homologous to GGA4 in a chromosomal signature unique to small New World ground doves.

## 1. Introduction

Birds have an enigmatic karyotype structured in two chromosomal groups distinguished by size-macrochromosomes (size from ~ 23 to 200 Mb) and microchromosomes (size from ~ 3 to 12 Mb), the latter representing the largest number of chromosomes in the karyotype [1,2,3]. This karyotypic structure is found in most avian species and is estimated to have been maintained since the diapsid common ancestor [4]. However, although a rare event, chromosomal rearrangements do occur and are often driven by breakpoint regions, usually associated with genomic features, including transposable elements and conserved noncoding elements [5].

The order Columbiformes (doves, pigeons and dodos) represents one of the oldest and most diverse extant lineages of birds, including approximately 300 living species [6,7,8], inhabiting a range of ecological environments in all continents except Antarctica [6]. The traditional taxonomic classification divides the Columbiformes into two families: Raphidae, which includes the dodo and the solitaire, and Columbidae, which includes doves and pigeons [6,9]. However, more recent phylogenetic studies support the inclusion of the dodo and the solitaire into the family Columbidae [7,8,10]. According to Pereira et al. [7], there are three major clades within the family Columbidae: clade A, containing genera from the Old and New World pigeons and doves, clade B includes the small Neotropical ground doves and clade C includes mostly genera found in the Old World (Afro-Eurasian and Australasian), the dodo and the solitaire. In this report, we analyze four genera belonging to clade A (*Geotrygon*, *Streptopelia* and *Patagioenas*) and clade B (*Columbina*).

Cytogenetic studies based on conventional staining has demonstrated that the diploid numbers in Columbiformes species range from 2n = 68 in *Uropelia campestris* to 2n = 86 in the genus *Geotrygon* [11,12,13,14,15]. Molecular cytogenetic characterization with *Gallus gallus* or *Zenaida auriculata* chromosome paints have been performed in five Columbidae species with a typical diploid number (2n = 76–80), and interchromosomal rearrangements were found in only two species [13,15,16]. *Streptopelia roseogrisea* has at least two chromosome fusions between chicken chromosomes 6, 7, 8 and 9, however, the particular chromosomes involved in these fusions could not be identified, due to the similarity in size and morphology of the derivative chromosomes and the use of only one fluorescent dye [13]. In *Leptotila verreauxi* a fusion was detected between chromosomes 6 and 7 [16]. Additionally, whole chromosome painting with *Leucopternis albicollis* probes in four Columbiformes species from different genera (*Leptotila*, *Zenaida*, *Columbina* and *Columba*) show a series of intrachromosomal rearrangements involving the ancestral chromosome 1 in all analyzed species [16]. The microchromosome organization has been analyzed only in *Streptopelia decaocto* and *Columba livia*, using bacterial artificial chromosome (BAC) probes for chicken chromosomes 10–28, and no rearrangements involving microchromosomes were detected [17].

Although substantial progress has been made in terms of understanding karyotype evolution within Columbiformes, it still remains poorly understood, especially with regard to the microchromosomes [13,14,16,17]. Therefore, the purpose of this study was to investigate the chromosome organization in six Columbidae species, representing four different genera, three of them with the typical diploid number (*Columbina*, *Patagioenas* and *Streptopelia*, 2n = 76) and one genus (*Geotrygon*, 2n = 86) with the highest diploid number for the family Columbidae. Furthermore, using chromosomal rearrangements as characters, we constructed a phylogenetic tree in order to compare with previous phylogenies based on molecular approaches. The results provide a comprehensive cytogenetic analysis of Columbiformes species based on molecular cytogenetics for macrochromosomes and microchromosomes and represent novel insights into chromosome evolution of Columbiformes.

## 2. Material and Methods

### 2.1. Cell Culture and Chromosome Preparation

The experiments were approved by the Ethics Committee on Animal Experimentation of Universidade Federal do Rio Grande do Sul (CEUA number 30750), and the samplings were authorized by the System of Authorization and Information in Biodiversity (SISBIO, number 33860-1 and 44173-1). Chromosome preparations were established from fibroblast cultures generated from skin biopsies according to Sasaki et al. [18]. The cells lines were established at 37 °C in Dulbecco’s Modified Eagle’s Medium (DMEM), supplemented with 15% fetal bovine serum, 2% Penicillin Streptomycin, and 1% L-glutamine. Chromosomes suspension were obtained after treatment with colcemid (1 h), hypotonic solution (0.075 M KCl, 15 min) and fixation with 3:1 methanol/acetic acid. The diploid number and chromosome morphology of each individual was determined in at least 20 metaphase chromosomes stained with Giemsa 10% in 0.07 M phosphate buffer, at pH 6.8. The species analyzed and methods performed are listed in Table 1.

### 2.2. Comparative Chromosome Painting

Chromosome specific paints of *Z. auriculata* (ZAU1-5 and Z) and *G. gallus* (GGA6-10) were generated from flow-sorted chromosomes and amplified by degenerate oligonucleotide-primed polymerase chain reaction (DOP-PCR). The paints were labeled with biotin-16-dUTP or digoxygenin dNTPs during secondary DOP-PCR amplification. Standard techniques were used for denaturation, hybridization, stringency washes and detection using Cy3-streptavidin for biotin-labeled probes or anti-digoxigenin FITC for digoxygenin labeled probes. Briefly, slides were pepsinized for 3 min, washed three times in 2 × SSC (5 min each), dehydrated in an ethanol series (2 min in 70% and 90%, and 4 min in 100% ethanol at room temperature) and incubated for 1 h at 65 °C. Probes were diluted in a hybridization buffer after incubation at 75 °C for 10 min and then pre-annealed at 37 °C for 30 min. Slides were denatured in 70% formamide/2 × SSC solution at 68 °C for 1 min and 20 sec, dehydrated through ethanol series and air-dried (2 min each in ice-cold ethanol 70%, 70%, 85% and 100% ethanol at room temperature). The probe mix was pipetted onto slides and covered with coverslips, sealed with rubber cement and incubated in a humidified chamber at 37 °C for 72 h. After that, they were washed for two times for 5 min in 50% formamide/2 × SSC followed by two times for 5 min in 2 × SSC at 40 °C, incubated three times in 4 × SSC Tween (0.05% Tween) for 5 min at room temperature. Chromosomes were counterstained with DAPI and analyzed using a Zeiss Axioplan2 fluorescence microscope and ISIS software (Metasystems).

Although we used some ZAU probes (ZAU1-5 and Z), the chromosomal comparisons were performed with the GGA homologous chromosomes [16], because most studies with comparative chromosome mapping in birds have concentrated on GGA probes, including the homology to the putative avian ancestral karyotype.

### 2.3. FISH with BAC Probes

Two BACs, selected from chicken or Zebra finch, were chosen for each of the microchromosomes GGA11-28 (except GGA16) according to O’Connor et al. [17] and applied to the selected species. The BAC clone isolation, amplification and labeling were performed following O’Connor et al. [17].

Chromosome preparations were fixed to slides and dehydrated through an ethanol series (2 min each in 2 × SSC, 70%, 85%, and 100% ethanol at room temperature). Probes were dissolved in a hybridization buffer (Cytocell) with chicken hybloc (Insight Biotech) and applied onto slides before sealing with rubber cement. The probe mix was simultaneously denatured on a 75 °C hotplate (2 min) prior to hybridization in a humidified chamber for 72 h at 37 °C. Slides were washed post-hybridization for 30 s in 2 × SSC w/0.05% Tween 20 at room temperature and counterstained with DAPI. The BACs FISH images were captured using an Olympus BX61 epifluorescence microscope with a cooled CCD camera and SmartCapture (Digital Scientific UK) system.

### 2.4. G-Banding

The G-banding was performed for ten Columbidae species with a combination of DAPI and propidium iodide [19] in order to detect intrachromosomal rearrangements not observed by chromosome painting. Images were captured by an Olympus BX61 epifluorescence microscope with a cooled CCD camera and SmartCapture (Digital Scientific UK) system. Afterward, the images were converted to grayscale using Corel Photo-Paint 2019.

### 2.5. Phylogenetic Analysis

A binary matrix of 28 discrete chromosomal rearrangements was conducted following Dobigny et al. [20]. A Maximum Parsimony (MP) tree was performed using PAUP 4.0b10 program [21]. The chromosomal rearrangements were established using the chicken chromosome painting (GGA 1–10) results obtained herein and from literature data [13,16] and the comparative chromosome analysis using G-banding. A heuristic search to find the most parsimonious tree(s) was performed using Tree Bisection Reconnection (TBR) branch-swapping. The bootstrap probability was performed with one thousand replicates, using chicken as an outgroup.

Additionally, the synapomorphic characters obtained in the MP analysis were also mapped onto a well-supported molecular phylogeny tree proposed by Pereira et al. [7], in order to verify which chromosomal characters give support to each branch in that topology. However, we only considered the chromosomal rearrangements detected by chromosome painting using chicken probes and G-banding since they were used in all Columbidae species analyzed so far.

## 3. Results

### 3.1. Macrochromosome Organization (GGA1-10)

**Figure 1:**
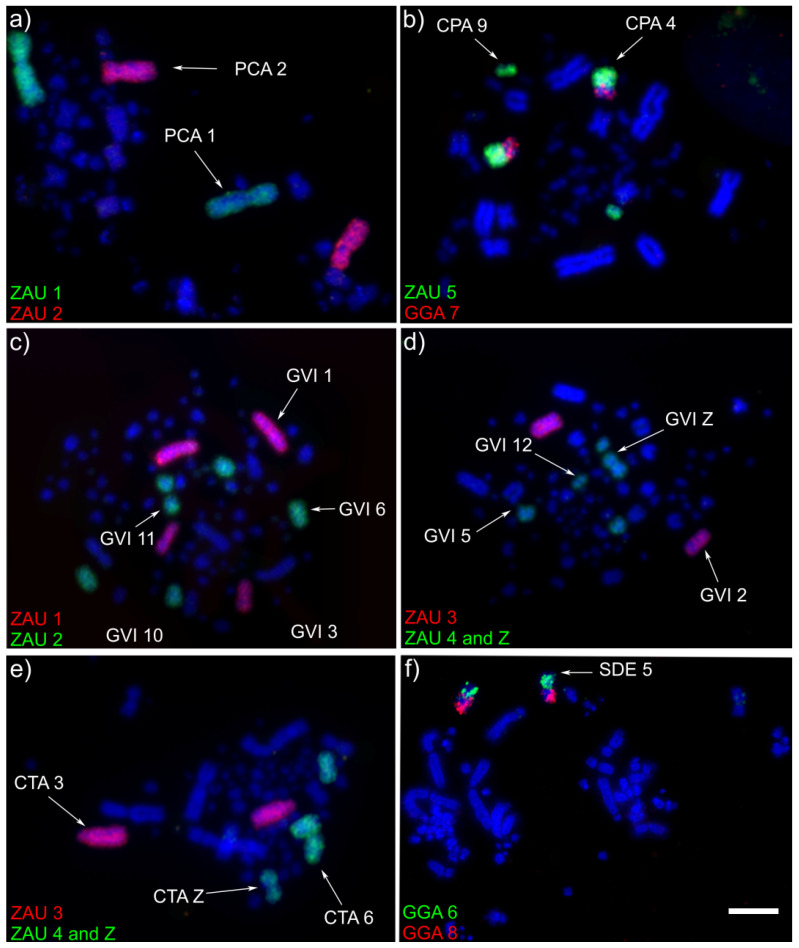
Representative FISH results using different sets of chromosome probes from *Gallus gallus* (GGA) and *Zenaida auriculata* (ZAU) probes on chromosomes of different Columbidae species: (**a**) *Patagioenas cayennensis* (PCA), (**b**) *Columbina passerina* (CPA), (**c**) *Geotrygon violacea* (GVI), (**d**) *Geotrygon violacea* (GVI), (**e**) *Columbina talpacoti* (CTA), (**f**) *Streptopelia decaocto* (SDE). The chromosome probes used are indicated on the left bottom, in green (fluoroscein labeled) or red (biotin-cy3 labeled). Scale bar 10 μm.

Chromosomal homologies were examined among six Columbidae species by whole chromosome painting with *G. gallus* and *Z. auriculata* chromosome-specific DNA paints. Representative results of FISH experiments are shown in Figure 1 and detailed below.

**Figure 2:**
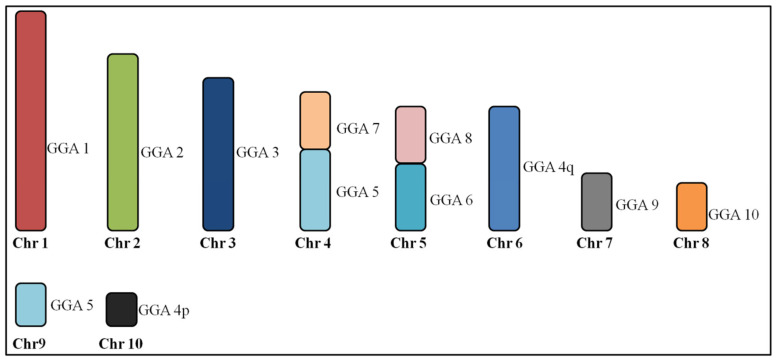
Homologous chromosomal segments of *Gallus gallus* (GGA) in *Columbina talpacoti* and *Columbina passerina* macrochromosomes as detected by fluorescence in situ hybridization (FISH) using GGA and *Zenaida auriculata* whole chromosome paints. Chr = Chromosome.

All GGA paints produced identical hybridization patterns in *Columbina talpacoti* (CTA) and *Columbina passerina* (CPA). GGA1–3, 9 and 10 paints each hybridized to a single chromosome pair, while paints GGA4 and 5 hybridized to two chromosomes pairs. One segment of GGA5 produced signals in the same chromosome pair as GGA7 (CPA4 and CTA4), while paints GGA6 and GGA8 hybridized to CPA5 and CTA5, revealing the occurrence of centric fusions. Figure 2 shows the homology between the *G. gallus* and *C. talpacoti* and *C. passerina*.

**Figure 3:**
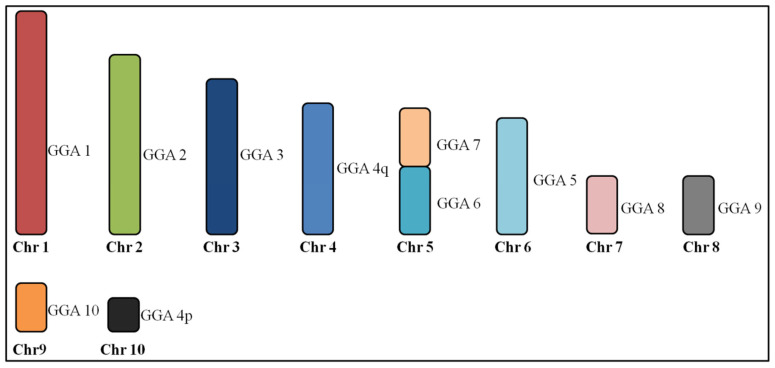
Homologous chromosomal segments of *Gallus gallus* (GGA) in *Patagioenas cayennensis* macrochromosomes as detected by fluorescence in situ hybridization (FISH) using GGA and *Zenaida auriculata* whole chromosome paints.

Hybridization of chicken paints GGA1–3 and 5, 8–10, each hybridized to a single chromosome pair in Patagioenas cayennensis (PCA). On the other hand, paint 4 hybridized to two chromosome pairs, while paints GGA6 and 7 were associated in one pair (PCA4), indicating the occurrence of centric fusion. Figure 3 shows the homology between the *G. gallus* and *P. cayennensis*.

**Figure 4:**
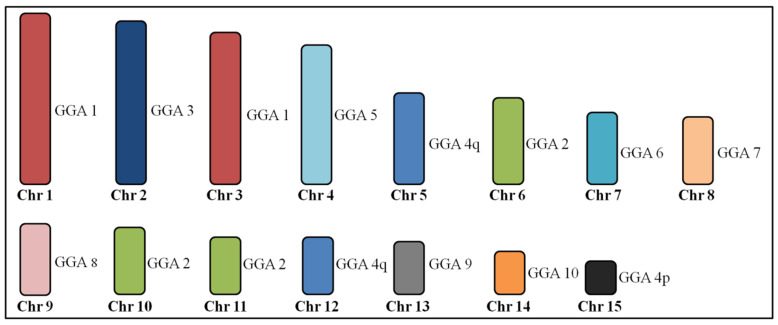
Homologous chromosomal segments of *Gallus gallus* (GGA) in *Geotrygon violacea* and *Geotrygon montana* macrochromosomes as detected by fluorescence in situ hybridization (FISH) using GGA and *Zenaida auriculata* whole chromosome paints.

The same hybridization pattern of the chicken probes was observed in *Geotrygon violacea* (GVI) and *Geotrygon montana* (GMO). GGA3, 5–10 probes each hybridized to a single chromosome pair, while paint GGA1 hybridized to two chromosome pairs, and paints GGA2 and 4 hybridized to three chromosomes pairs. Figure 4 shows the homology between the *G. gallus* and *G. violacea* and *G. montana*.

**Figure 5:**
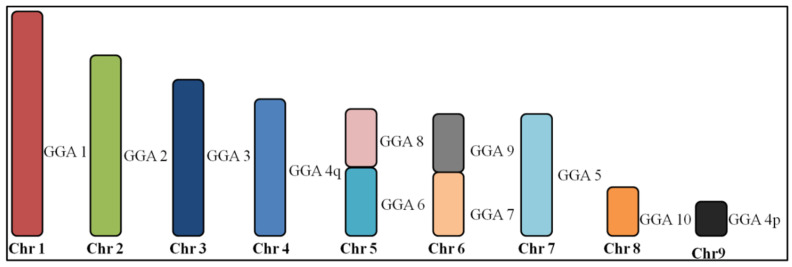
Homologous chromosomal segments of *Gallus gallus* (GGA) in *Streptopelia decaocto* macrochromosomes as detected by fluorescence in situ hybridization (FISH) using GGA and *Zenaida auriculata* whole chromosome paints.

In *S. decaocto* (SDE), chicken chromosome paints 1–3 and 5, each hybridized to a single chromosome pair, while paints GGA4 hybridized to two chromosome pairs. Chicken paints GGA6–9 confirmed that these chromosomes are involved in two fusions, as observed in *Streptopelia roseogrisea* [12]. However, here the fusions involving GGA6/8 and GGA7/9 were identified. It is likely that these fusions are also present in *S. roseogrisea*, since both have similar karyotypes and are considered sister species. Figure 5 shows the homology between the *G. gallus* and *S. decaocto*.

### 3.2. Microchromosome Organization (GGA11-28, Except GGA16)

**Figure 6:**
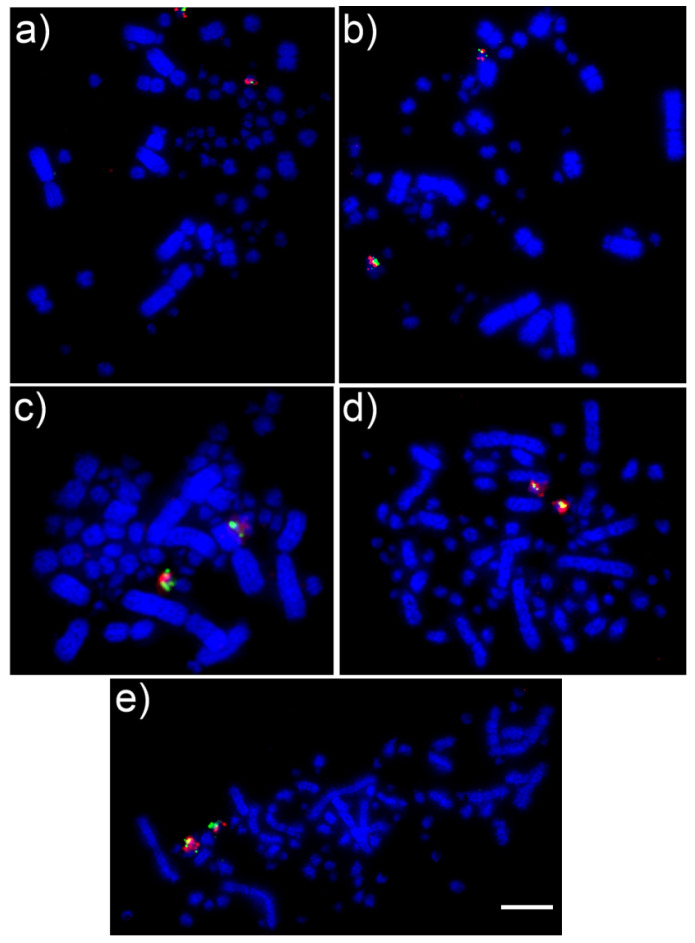
FISH using BACs for chicken chromosome 26 (CH261-186M13 FITC and CH261-170L23 Texas Red) on chromosomes of different Columbidae species revealing no evidence of interchromosomal rearrangements: (**a**) *Columbina talpacoti*, (**b**) *Patagioenas cayennensi,* (**c**) *Columbina passerina*, (**d**) *Geotrygon violacea*, (**e**) *Geotrygon montana*. Scale bar 10 μm.

Results for *C*. *talpacoti*, *C*. *passerina*, *G*. *violacea*, *G*. *montana* and *P*. *cayennensis* provide no evidence of interchromosomal rearrangements in the microchromosomes in any of the five species here analyzed. All chicken microchromosome BACs GGA11-28 (except GGA16) were efficiently cross-hybridized to chromosomes of all five Columbidae species, revealing the ancestral microchromosomal pattern, similar to results observed in *S*. *decaocto* and *C*. *livia*, previously performed by O’Connor et al. [17]. Examples of the BACs FISH results are demonstrated in Figure 6 for chromosome 26 for all species.

### 3.3. G-Banding

G-banding patterns generated with a combination of DAPI and propidium iodide (Supplementary Figure S1) in ten Columbidae species and chicken were used to detect chromosomal homology between these species after visual inspection (Supplementary Table S1).

### 3.4. Phylogenetic Analysis

**Figure 7:**
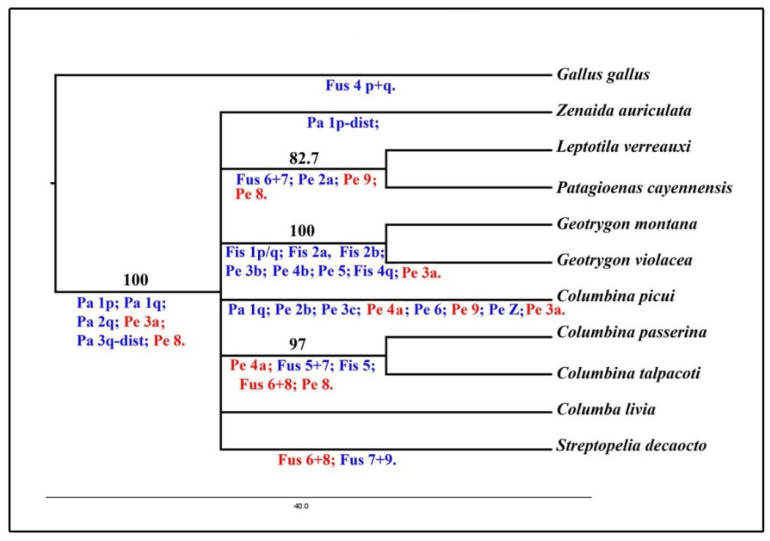
Phylogenetic analysis of maximum parsimony using PAUP based on chromosome rearrangements present in Columbidae species according to results obtained by whole chromosome painting with *Gallus gallus* and *Zenaida auriculata* probes and G-banding. All rearrangement characters are mapped a posteriori and are shown below the branches. Characters in red indicate chromosome rearrangements shared for more than one branch (homoplasic characters). Black numbers above the branch indicate the bootstrap value with one thousand replicates.

The data on chromosome painting and G-banding obtained from ten species of Columbiformes and the outgroup (*G. gallus*) were used to generate a matrix with 28 discrete chromosome characters (Supplementary Table S1 and Supplementary Figure S2). Only macrochromosomes were used in this analysis since no interchromosomal rearrangement involving microchromosomes was observed in the Columbidae species. The MP analysis resulted in 5 most parsimonious trees (tree length = 35, consistence index = 0.800, retention index = 0.6957 and homoplasy index = 0.200) (Supplementary Figure S3). Overall, 16 characters were parsimoniously informative. Despite the fact that Columbiformes correspond to a monophyletic group supported by six synapomorphies and a bootstrap value of 100, the species form an unresolved polytomy (Figure 7). However, three monophyletic clades, supported by high bootstrap values, are observed among this politomy: *L. verreauxi* and *P. cayennensis*, *G. montana* and *G. violacea*, *C. passerina* and *C. talpacoti*.

## 4. Discussion

In this study, we have described the karyotypes of six Columbidae species (*C*. *talpacoti*, *C*. *passerina*, *P*. *cayennensis*, *G*. *violacea*, *G*. *montana* and *S*. *decaocto*) using *G. gallus* and *Z. auriculata* chromosome painting and have performed an integrative analysis with previously published maps of another four species (*Z*. *auriculata*, *L*. *verreauxi*, *C*. *livia* and *C*. *picui*) [16]. The whole chromosome painting performed here demonstrated that each Columbidae species showed at least one interchromosomal rearrangement involving macrochromosomes when compared with *G*. *gallus*, as observed previously in *L*. *verreauxi*, but in contrast to *Z*. *auriculata*, *C*. *livia* and *C*. *picui*, in which no interchromosomal rearrangements were found. However, the G-banding results (10 species) indicated that intrachromosomal rearrangement is the main driver of chromosome evolution in Columbidae species, being evident in all Columbidae species, even in species without interchromosomal rearrangements (i.e., *Z*. *auriculata*, *C*. *livia* and *C*. *picui*). On the other hand, we observed a high degree of genome stability in the microchromosomes of all these species.

### 4.1. Macrochromosome Organization

Typical karyotypes are found in *C*. *talpacoti* (2n = 76), *C*. *passerina* (2n = 76), *P*. *cayennensis* (2n = 76) and *S*. *decaocto* (2n = 76), since 61.3% of birds have 2n = 76–82. However, atypical karyotypes are found in *Geotrygon* species because only 1.2% of birds have 2n = 86 [22]. There are clear differences in chromosomal morphologies among the *Geotrygon* species in relation to the other Columbidae species [15]. While Columbidae species generally have between five and eight biarmed chromosomes, all the autosomal chromosomes of *Geotrygon* species are telocentric [15]. The comparative chromosome painting performed in this study has brought to light the extent of evolutionary karyotype organization among the different species of the Columbidae family.

Whole chromosome paints derived from GGA1-10 produced identical results in *C*. *talpacoti* and *C*. *passerina*, including a fusion between GGA6/GGA8. Interchromosomal fusions are exceptionally rare in birds, and evidence of fusion between GGA6/GGA8 has only been previously described in three not-closely related species, *Tetrao urogallus* (Galliformes), *Falco columbarius* (Falconiformes) and *Opisthocomus hoazin* (Opisthocomiformes) [23,24,25], hence, this fusion in *C*. *talpacoti* and *C*. *passerina* likely occurred in their common ancestor. This hypothesis is reinforced by the absence of this fusion in other species of the same genus, *C. picui* [16]. Apart from the GGA6/GGA8 fusion, we have found the fission of ancestral chromosome 5 (GGA5) and a fusion between a segment of GGA5 with GGA7 in *C*. *talpacoti* and *C*. *passerina*. These two rearrangements were also not found in *C*. *picui*, reinforcing the phylogenetic proximity between *C*. *talpacoti* and *C*. *passerina* [26]. To our knowledge, the association between GGA5 and GGA7 is a rare event in birds, having been reported only in *Melopsittacus undulatus* (Psittaciformes) in this species, however, these chromosomes are also fused with segments from other chromosomes [27].

The chromosome complement of *P*. *cayennensis* is derived from the ancestral karyotype (2n = 80) by a centric fusion between GGA6/7, which was recently found in *L*. *verreauxi* [16]. Although this rearrangement may have a common origin in both species, we cannot discard the possibility of convergent evolution, since it seems to be one of the most common associations in birds, being found also in Galliformes [23], Gruiformes [28], Strigiformes [29], Trogoniformes [30] and Psittaciformes [27,31].

The karyotypes of *G*. *violacea* and *G*. *montana* are derived from the putative ancestral avian karyotype by fissions of the chromosomes 1, 2 and 4. This fact explains the higher diploid number observed in *G*. *violacea* and *G*. *montana*, with 2n = 86, compared to other Columbidae species (around 2n = 76).

Results of chromosome painting shown here demonstrate that the fusions between GGA6/8 and GGA7/9 are present in *S*. *decaocto*, and are probably also present in *S. roseogrisea*, because both species are considered to be sister species [26] and present similar karyotypes [13]. Besides that, the fusion between GGA7/GGA9 in *S*. *decaocto* was detected for the first time in any avian species and may be a synapomorphic trait for the genus *Streptopelia*. In contrast to *S*. *roseogrisea*, we did not find the fusion between GGA4q and GGA4p in *S*. *decaocto*.

### 4.2. Conservation of Microchromosome Organization

No interchromosomal rearrangements involving microchromosome pairs GGA11-28 (GGA16 not tested) were found in the species analyzed, as previously observed in two other Columbidae species, *C*. *livia* and *S*. *decaocto* [17]. The lack of interchromosomal rearrangement observed in the Columbidae species corroborates the possible evolutionary advantage of retaining this pattern of microchromosome organization, as previously proposed [17].

### 4.3. Phylogenetic Relationships in the Family Columbidae

Our chromosomal phylogeny gives strong support for the monophyly of doves and pigeons, with high bootstrap value (Figure 7). This ancient branch received 100% of bootstrap support, having six chromosome characters supporting the basal position for the group (Figure 7). This data corroborates previous molecular phylogenies that also demonstrate the monophyly of the group with strong support [7,8]. Despite the chromosome similarities of Columbiformes and the outgroup *G. gallus*, the main rearrangements in Columbidae species are intrachromosomal rearrangements, as revealed by G-banding.

However, we could not resolve the topology of the Columbidae family using chromosomal rearrangements. After the divergence of the basal branch of the Columbiformes, we found a polytomy, in which *Z*. *auriculata*, *C*. *picui*, *C*. *livia* and *S*. *decaocto* do not group with any other species. This is a commonly observed scenario in chromosomal phylogenies, especially in birds in which the karyotype changes occur at a low rate when compared with other groups [32,33]. Furthermore, the diversification of pigeon and doves occurred in the late Oligocene and continued to diversify into Miocene around 24.7 Mya [8], and hence the common ancestor of the main lineages may not have been present long enough to accumulate chromosome differences detectable by G-banding or chromosome painting.

On the other hand, three clades, formed by *L*. *verreauxi* and *P*. *cayennensis*, *C*. *talpacoti* and *C*. *passerina,* and *G*. *montana* and *G*. *violacea* were observed in our consensus tree (Figure 7). Within the Holarctic doves, *Geotrygon* species (*G*. *violacea* and *G*. *montana*) presented the most derived karyotypes, resulting in seven synapomorphies supporting this clade (fission on GGA1, two fission on GGA2, pericentric inversion on GGA 3, pericentric inversion on GGA 4, pericentric inversion on GGA 5 and GGA4q fission). Another clade was formed by *L*. *verreauxi* and *P*. *cayennensis*, supported by three derived characters (two synapomorphies, pericentric inversion on GGA2 and GGA9 and one homoplasy - fusion between GGA6/7). This clade is unusual when we compare it with consensual topologies based on DNA sequence, which usually support the close affinities of the genus *Geotrygon*, *Zenaida* and *Leptotila* allied with another branch containing genus *Patagioenas*, *Streptopelia* and *Columba* [7,8,10].

In the three species analyzed of New World ground doves, only two species group together, with five characters supporting *C*. *talpacoti* and *C*. *passerina* as sister groups (pericentric inversion on GGA 4 and 8, fusion of GGA 6/8 and GGA5/7, and fission of GGA5). The species *C. picui* does not group with other species of the genus, probably due to the loss of some chromosome characters or its basal position in previous phylogenies [34]. Hence, the fusion between GGA 5/7 and the fission in GGA5 may have arisen only in the common ancestor of *C*. *talpacoti* and *C*. *passerina*. The pericentric inversion on GGA4 is shared among *Columbina* species analyzed herein and could be a synapomorphy for the genus. However, despite the highly reshuffled karyotype of *C*. *picui* and due to the accumulated homoplasic characters, the affinity of the genus *Columbina* was not retrieved through parsimony analyses. Hence, future studies are necessary to reconstruct the chromosomal history of Columbiformes using more taxa (including closely related outgroups) and other methodologies in order to test conflicting homologies and to increase the number of informative characters.

**Figure 8:**
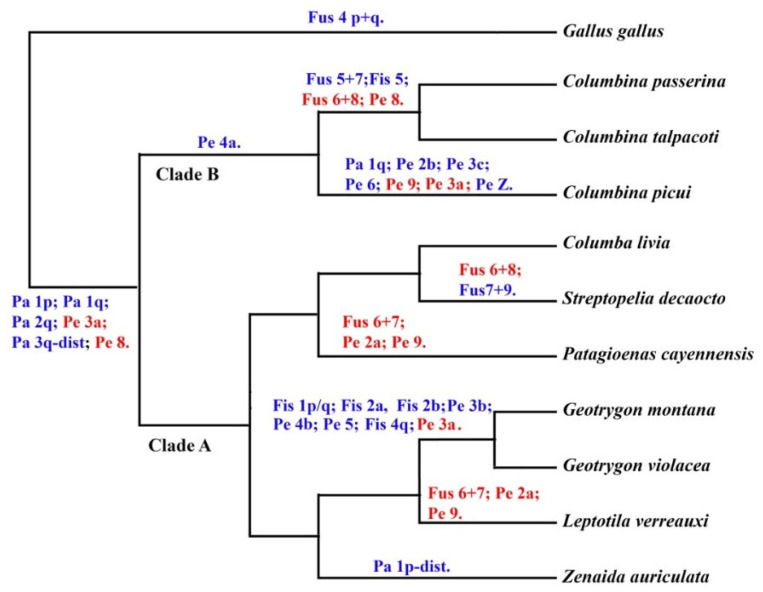
Chromosomal rearrangements in Columbidae species detected by chromosome painting using *Gallus gallus* and *Zenaida auriculata* probes and G-banding plotted in a molecular phylogeny [2]. Characters in red indicate chromosome rearrangements shared for more than one branch (homoplasic characters). Published chromosome painting data from Kretschmer et al. [10] and the data obtained in this study were used for this figure.

An interesting approach in the investigation of the karyotype evolution of the Columbidae family is plotting the chromosomal rearrangements in a well-resolved phylogeny, such as proposed by Pereira et al. [7], using nuclear and mitochondrial DNA genes (Figure 8). For instance, these authors recovered three major clades within the Columbiformes: clade A, with genera from Old and New World pigeons and doves, clade B with small New World ground doves and clade C with genera found mostly in the Old World. However, chromosomal characters did not give support to clade A, although data from other Columbidae species are still required for a more thorough appreciation of the role of chromosomal rearrangements in this clade. On the other hand, a pericentric inversion in the chromosome homologous to GGA4 (character 15) supported clade B, which includes a group of small ground dove species.

The fusion between GGA6/GGA8 may have a common origin in *C*. *talpacoti* and *C*. *passerina*, since this fusion is not common in birds [2,25]. However, if the phylogeny proposed by Pereira et al. [7] is correct, this fusion is likely to be a convergent chromosome rearrangement in *S. decaocto* and in *C*. *talpacoti* and *C*. *passerina*. In the same way, the pericentric inversion on GGA2 and GGA9 and the fusion between GGA6/7 also would represent a convergent chromosome rearrangement in *L*. *verreauxi* and *P*. *cayennensis*.

## 5. Conclusions

In summary, out of 10 Columbidae species analyzed by chromosome painting so far (including the six species analyzed here), we present the first report of chromosome fission in Columbidae species, i.e., *C*. *talpacoti*, *C*. *passerina*, *G*. *violacea* and *G*. *montana*. Chromosomal fusions were observed in *C*. *talpacoti*, *C*. *passerina*, *P*. *cayennensis* and *S. decaocto*, and previously in *L. verreauxi* and *S. roseogrisea*. In view of the conservation of microchromosome organization in the Columbidae species, we suggest that the main forces in the chromosome evolution in Columbidae species are interchromosomal rearrangements involving macrochromosomes and also intrachromosomal rearrangements, as observed in all Columbidae species analyzed here by G-banding.

The comparisons of the chromosome organizations presented here allow us to speculate on the process of karyotype evolution and the overall picture of the phylogeny in several members of the family Columbidae. Although *C*. *talpacoti*, *C*. *passerina*, *P*. *cayennensis* and *S. decaocto* have undergone interchromosomal rearrangements in the macrochromosomes, the typical ‘avian-like’ karyotype (~ 80 chromosomes) has been maintained in these species. However, due to interchromosomal fissions in the macrochromosomes, atypical karyotypes (2n = 86) were found in *G*. *violacea* and *G*. *montana*. Nevertheless, taking into account the possibility of a high percentage of convergent chromosome rearrangement observed in Columbidae species, we could not resolve the phylogeny of Columbidae members using chromosome rearrangements. Considering the high frequency of intrachromosomal rearrangements in avian genome evolution [35] and in Columbidae [16,36], we believe that the key to reconstructing the evolutionary history of the Columbiformes must be in the intrachromosomal rearrangements. Hence, future strategies that expand the ability to detect smaller rearrangements in macrochromosomes, for example, using BACs probes to each macrochromosomes, are needed to increase our knowledge about karyotype evolution and to resolve the phylogeny using chromosome rearrangements. Furthermore, molecular cytogenetic studies in species from clade C are necessary to improve our knowledge about the direction of chromosomal changes in all three clades in the Columbidae family.

## Figures and Tables

**Table 1:** List of Columbidae species and cytogenetics methods performed in this study. WCP = whole chromosome probes.

Species	Number of Individuals/Sex	2n	Wcp	Micro BACs	G-Banding
*Columbina talpacoti*	2 M	76	Present study	Present study	Present study
*Columbina passerina*	1 M	76	Present study	Present study	Present study
*Columbina picui*	1 M and 1 F	76	[16]	-	Present study
*Columba livia*	1 M	80	[16]	[17]	Present study
*Geotrygon montana*	1 M	86	Present study	Present study	Present study
*Geotrygon violacea*	1 F	86	Present study	Present study	Present study
*Leptotila verreauxi*	2 M	78	[16]	-	Present study
*Patagioenas cayennensis*	2 M	76	Present study	Present study	Present study
*Streptopelia decaocto*	1 F	76	Present study	[17]	Present study
*Zenaida auriculata*	2 M	76	[16]	-	Present study

M = male, F = female.

## Supplementary Materials

The following are available online at https://www.mdpi.com/2073-4425/11/6/632/s1, Figure S1: G-banded macrochromosomes of *Gallus gallus* (GGA), ZAU, *Zenaida auriculata*; CPA, *Columbina passerina*; CTA, *Columbina talpacoti*; CPI, *Columbina picui*; LVE, *Leptotila verreauxi*; CLI, *Columba livia*; SDE *Streptopelia decaocto*; PCA, *Patagioenas cayennensis*; GMO, *Geotrygon montana*, and; GVI, *Geotrygon violacea*. Figure S2: G-banded macrochromosomes of *Gallus gallus* (GGA), ZAU, *Zenaida auriculata*; CPA, *Columbina passerina*; CTA, *Columbina talpacoti*; CPI, *Columbina picui*; LVE, *Leptotila verreauxi*; CLI, *Columba livia*; SDE *Streptopelia decaocto*; PCA, *Patagioenas cayennensis*; GMO, *Geotrygon montana*, and; GVI, *Geotrygon violacea* indicating the chromosome characters used in the phylogenetic analysis. An asterisk indicates a heteromorphism in GMO1 and 3. Figure S3: The five most parsimonious tree (A–E) resulted in the maximum parsimony analysis (MP) using PAUP based on chromosome rearrangements present in Columbidae species according to results obtained by whole chromosome painting with *Gallus gallus* and *Zenaida auriculata* probes and G-banding. All rearrangement characters were mapped a posteriori and are shown below the branches. Characters in red indicate chromosome rearrangements shared for more than one branch (Homoplasic characters). Table S1: Binary Matrix of chromosome characters obtained by chromosome painting and G-band. *Gallus gallus* (GGA), ZAU, *Zenaida auriculata*; CPA, *Columbina passerina*; CTA, *Columbina talpacoti*; CPI, *Columbina picui*; LVE, *Leptotila verreauxi*; CLI, *Columba livia*; SDE *Streptopelia decaocto*; PCA, *Patagioenas cayennensis*; GMO, *Geotrygon montana*, and; GVI, *Geotrygon violacea*.
